# Preparation and Application of Porous Metallic Glasses via Aging-Assisted Ultrasonic Vibration and Compression

**DOI:** 10.3390/ma18245484

**Published:** 2025-12-05

**Authors:** Jiaqing Lin, Heting Zhang, Zhe Chen, Jihan Jiang, Xingran Zhao, Xiaodi Liu, Wenqing Ruan, Jiang Ma

**Affiliations:** 1 Shenzhen Key Laboratory of High Performance Nontraditional Manufacturing, College of Mechatronics and Control Engineering, Shenzhen University, Shenzhen 518060, China; 2310295178@email.szu.edu.cn (J.L.); 2110292036@email.szu.edu.cn (H.Z.); 243101022@csu.edu.cn (Z.C.); 2310295138@email.szu.edu.cn (J.J.);; 2 School of Materials Science and Engineering, Central South University, 932 South Lushan Road, Changsha 410083, China

**Keywords:** metallic glasses, dealloying, oxygen evolution reaction, hydrogen evolution reaction

## Abstract

The quest for enhanced energy efficiency is inextricably linked to advancements in energy storage and conversion, with porous metallic glasses (MGs) serving as catalysts that hold significant potential in this area. In this study, we report the preparation of uniform porous structures by aging-assisted ultrasonic vibration (AAUV). The results indicate that ultrasonic treatment effectively enhances the energy state while preserving the amorphous structure of Zr_62_Cu_15.5_Ni_12.5_Al_10_ MGs. The results demonstrate that UV treatment effectively elevates the energy state while maintaining the amorphous structure. Electrochemical tests reveal significantly improved chemical activity after UV treatment, with a reduced corrosion potential and over 200-fold increase in electrochemical surface area after dealloying. The dealloyed UV-treated samples develop uniform porous structures with Cu-enriched zones, exhibiting exceptional catalytic performance in alkaline media (oxygen evolution reaction: 350 mV, hydrogen evolution reaction: 163 mV), comparable to commercial catalysts. This work provides new insights into developing high-performance MGs through energy-state engineering.

## 1. Introduction

Metallic glasses (MGs) have garnered significant attention as engineering materials due to their unique physical and chemical properties [1,2,3,4]. However, prolonged usage or storage often leads to structural relaxation [5,6,7,8]. This time-dependent evolution is known as the aging effect, which originates from the intrinsic thermodynamic metastable stability of these amorphous systems, allowing the initial state to transition toward a more stable state over time [9,10,11]. The properties of MGs tend to deteriorate markedly with aging. To address this, researchers have explored various methods to restore and enhance the performance of aged MGs, among which ultrasonic vibration (UV) treatment has emerged as a highly promising technique [10,12,13,14,15].

UV loading can induce atomic rearrangement within the amorphous structure, thereby altering its microstructure and energy state [16,17,18,19], transitioning the alloy from a low-energy (relaxed) state to a higher-energy state [10,20,21]. Consequently, the treated alloy exhibits not only improved mechanical properties (e.g., plasticity) but also significant modifications in its electrochemical behavior. Notably, the rejuvenated alloys exhibited enhanced electrochemical performance, including improved reaction kinetics and increased hydrogen evolution reaction (HER) and oxygen evolution reaction (OER) activities. These findings underscore the dual functional benefits of UV treatment—structural recovery and catalytic activation—making it a viable strategy for extending the service life and broadening the applications of MGs. Although electrochemical performance has been improved, there is still much room for growth.

An alternative approach to improve catalytic performance involves fabricating porous materials to increase their specific surface area. Porous metals exhibit three key advantages: high surface-to-volume ratio, excellent electrical and thermal conductivity, and remarkable mechanical stability. These characteristics make them particularly attractive for applications in catalysis, sensing devices, filtration systems, and biotechnology [22,23,24,25,26]. Porous materials can be fabricated through various methodologies, each exhibiting distinct advantages and limitations. The pore-forming agent addition approach involves incorporating sacrificial templates during material synthesis, which are subsequently removed through thermal decomposition to generate porous architectures. While this method offers operational simplicity, precise control over pore size distribution remains challenging. Alternatively, the particle packing technique utilizes random or ordered assembly of particulate matter to create porous networks. However, this method provides limited control over pore morphology and spatial arrangement. More advanced fabrication techniques include powder extrusion-based 3D printing (PEP), which enables layer-by-layer deposition of ceramic-binder composites to construct designed porous structures. Although additive manufacturing provides exceptional design flexibility and rapid prototyping capabilities, current limitations include substantial equipment costs and incomplete process optimization. Dealloying, which is a technique for fabricating porous materials by selectively dissolving non-precious metal elements [18,19,27]. During this process, the more noble residual elements undergo structural reorganization [28,29,30,31,32,33]. Compared with crystalline materials, MGs offer distinct advantages for dealloying due to their disordered glassy structure [34]. Due to the absence of crystal defects such as dislocations, MGs can theoretically form a more uniform porous structure.

However, due to their unique disordered structure, MGs exhibit excellent corrosion resistance, which poses a significant challenge to the traditional dealloying process. Meanwhile, there are strong correlations between corrosion current density and both metal-metal bond strength and metal-oxygen bond strength. An amorphous structure can facilitate the formation of denser passive film, and thus superimpose a positive influence on alloys that inherently exhibit corrosion resistance [35]. Therefore, the process of preparing porous MG materials through alloying remains a significant challenge.

In this study, we report that Aging-Assisted Ultrasonic Vibration (AAUV) treatment facilitates the formation of a uniform porous structure during the dealloying process of MGs. This energy-state modulation engineering enhances atomic mobility and chemical activity in the UV treatment samples, effectively lowering the kinetic barriers for selective dissolution during dealloying. Moreover, the dealloyed porous samples prepared by this method exhibit remarkable electrochemical performance. Under alkaline conditions, these materials achieve outstanding catalytic metrics with OER and HER overpotentials of merely 350 mV and 163 mV at 10 mA/cm^2^ current density, respectively, representing superior performance among non-precious metal catalysts. These findings position UV rejuvenation as a promising technique for fabricating and modifying porous MGs, with significant potential for widespread application.

## 2. Materials and Methods

In this work, the raw metal particles Zr (99.999 at.%), Cu (99.999 at.%), Ni (99.999 at.%), and Al (99.999 at.%) were purchased commercially. After ultrasonically cleaning with ethanol, and then in a high-purity argon atmosphere, the materials were cast using a copper mold to produce 2 mm round bars of the Zr_62_Cu_15.5_Ni_12.5_Al_10_ (at.%) alloy (hereafter referred to as Zr62). The rods were placed in an annealing furnace and annealed at 653 K (*T*_g_) for 10 h. To prevent oxidation during annealing, it was placed inside a quartz tube and finally cooled to room temperature in air.

The ultrasonic sonotrode is made of cemented carbide (TC4 titanium alloy) and integrates with the booster and transducer, whose primary function is to convert electrical signals into high-frequency vibrations. The ultrasonic sonotrode applies mechanical vibrations perpendicular to the sample at a preset pressure. The frequency is 20,000 Hz (frequency tolerance range ± 500 Hz). The cylindrical samples used in this study have a diameter of 2 mm and a length of 4 mm. The equipment features two control modes: time-control and energy-control. In this work, we employed the energy-control mode, the stress being measured by a load cell positioned beneath the sample. The maximum stress during the ultrasonic loading process is 25 MPa, with a loading duration of approximately 0.45 s.

Analysis of phases was conducted using X-ray diffraction (XRD, Rigaku MiniFlex 600, Tokyo, Japan) with Cu Kα radiation. The X-ray wavelength was 0.154 nm, the rated power was 600 W, and the scanning angle range was 20° to 80°. Transmission electron microscopy (TEM, FEI F30, Hillsboro, OR, USA) was employed to characterize the microstructure of the samples. All TEM samples in the present study were prepared using focused ion beam technology, and the type of ion source is a liquid Ga ion source. The nanoindentation tests were performed using a Hysitron TI 950 TriboIndenter (Bruker Corporation, Los Angeles, CA, USA) equipped using a Berkovich triangular pyramid indenter with a tip radius of 20nm. And the thermal properties of the samples were determined using differential scanning calorimetry (DSC, Perkin Elmer DSC8000, Waltham, MA, USA) with a cooling/heating rate of 20 K min^−1^.

Electrochemical workstation (CHI 760E, Shanghai Chenhua, Shanghai, China) was employed under standard three-electrode configuration at room temperature. Pt foil served as the counter electrode, and reference electrode was a silver chloride electrode. Dealloying treatment was conducted (2 mol/L HCl, −0.232 V vs. RHE Ag/AgCl for 2 h). The resulting dealloying samples were then thoroughly rinsed with deionized water and ethanol. Concurrently, the electrocatalytic activity for HER and OER was evaluated under alkaline conditions (1 M KOH). Electrochemical impedance spectroscopy (EIS) was performed with frequencies ranging from 0.01 Hz to 10^6^ Hz with an amplitude of 5 mV. The measurement potential of the reversible hydrogen electrode (RHE) is determined according to the Nernst equation E*_RHE_* = E*_Ag/AgCl_* + 0.059 × pH + 0.197 Tafel plots were fitted to the Tafel equation: *η* = *β* log *j* + α, where *η* is the half-wave potential, *β* is the Tafel slope, *j* is the current density, and *α* is the Tafel intercept relative to the exchange current density *j*.

## 3. Results and Discussion

### 3.1. Structural Evolution and Property Changes of Samples After Different Treatments

**Figure 1:**
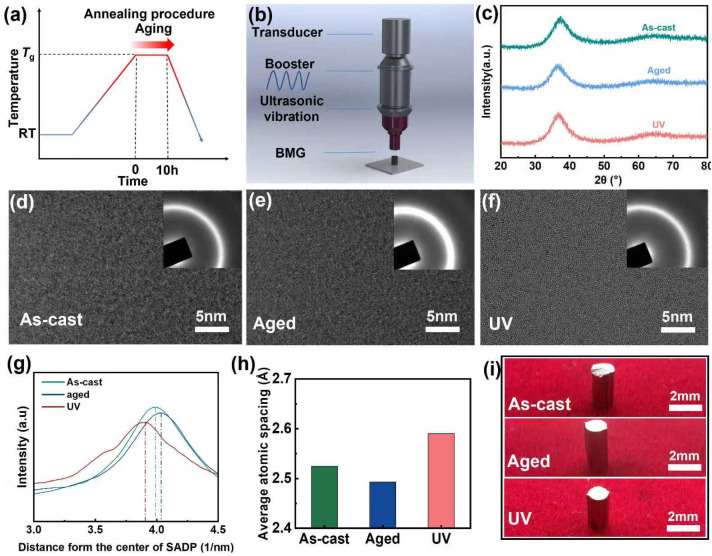
Property changes of Zr62 aged MGs and UV loading process. (**a**) Schematic diagram of the annealing process of MGs. (**b**) Schematic diagram of the UV loading process of MGs. (**c**) XRD patterns for samples of As-cast, Aged and UV. (**d**–**f**) HRTEM images and corresponding SAED images of the samples. (**g**) Diffraction ring radius obtained by SAED in (**d**–**f**). Dashed lines are used to emphasize the position of the highest point on the curve. (**h**) Average atomic spacing obtained by diffraction ring radius. (**i**) Photos of diferent processed samples.

In this study, MG rods with a nominal composition of Zr_62_Cu_15.5_Ni_12.5_Al_10_ (Zr62) were prepared through arc-melting followed by rapid cooling. To accelerate the aging process, the as-cast rods were vacuum-sealed and annealed at *T*_g_ for 10 h before being cooled to room temperature, yielding aged samples (Figure 1a) [36,37]. Subsequently, UV treatment with an energy input of 100 J was applied to the aged samples to obtain rejuvenated specimens (Figure 1b).

Structural characterization by X-ray diffraction (XRD, Figure 1c) and high-resolution transmission electron microscopy (HRTEM, Figure 1d) confirmed the preserved amorphous nature of both aged and rejuvenated samples. Quantitative analysis of diffraction ring radii through intensity profiling (Figure 1g) and subsequent peak position comparison (Figure 1h) demonstrated distinct atomic-scale structural evolution: aged samples exhibited reduced atomic spacing while UV-treated samples showed expanded interatomic distances compared to the as-cast state. These observations align with established literature correlating atomic packing density with energy states—tighter atomic packing corresponds to lower energy states with reduced chemical activity, whereas expanded atomic configurations represent higher energy states with enhanced reactivity [10]. The significant atomic spacing expansion in UV-treated samples confirms their transition to higher energy states through UV loading. Optical microscopy images (Figure 1i) show that the as-cast, aged, and UV samples exhibit essentially no macroscopic changes.

**Figure 2:**
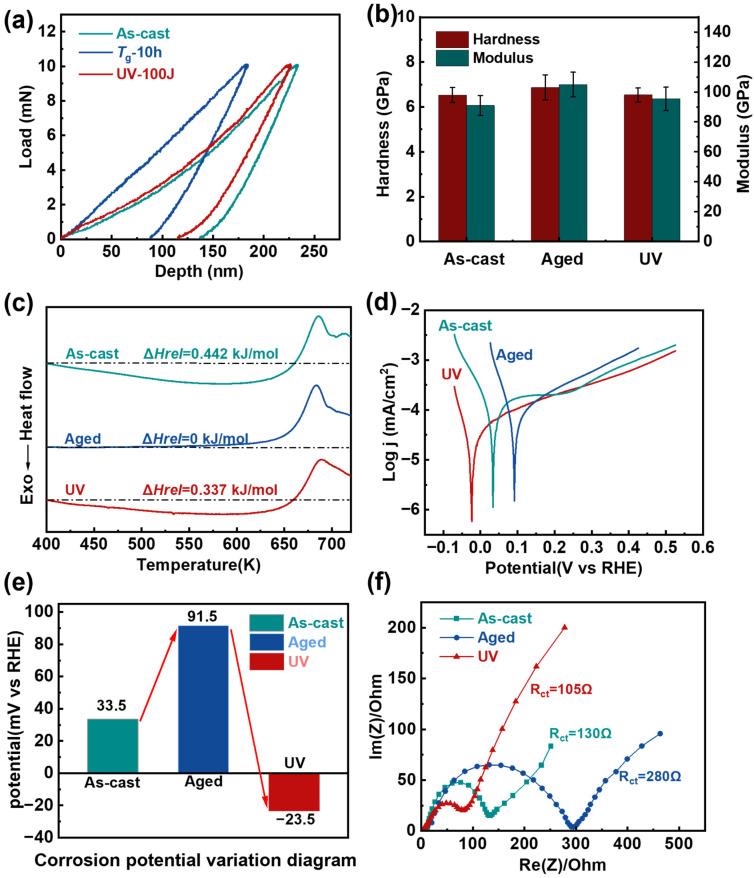
Property evolution of the samples undergoing different treatments. (**a**) Indentation depth corresponds to changes in hardness and modulus of nanoindentation. (**b**) Comparison of the hardness and modulus between the three samples. The error bars represent the standard deviations of the measured values (**c**) DSC curves of the three samples. Dashed lines are used to highlight the magnitude of the Δ*H*_rel_ on the DSC curves. (**d**) Corrosion curves of the three samples. (**e**) Corrosion potential change of three samples (**f**) Nyquist plots of three samples from 0.01 Hz to 10^6^ Hz for those samples.

To further investigate the atomic-scale structural evolution among the as-cast, aged, and UV samples, nanoindentation tests were systematically performed. The load–displacement curves (Figure 2a) reveal distinct mechanical responses: the aged sample exhibited significantly reduced indentation depth compared to the as-cast specimen, while UV treatment effectively restored the indentation depth to levels approaching the initial as-cast state. Complementary analysis of the nanoindentation-derived mechanical properties (Figure 2b) demonstrated that thermal aging treatment increased both hardness and elastic modulus relative to the as-cast condition. After UV treatment is applied to the aged samples, their hardness and modulus decrease to a certain extent. These mechanical property variations provide strong corroborating evidence for the atomic spacing changes observed through structural characterization techniques, as the measured softening behavior is consistent with expanded interatomic distances in the rejuvenated material.

To explore the changes in energy states, we conducted DSC measurements on all three sample states (Figure 2c). The DSC results revealed a systematic evolution of relaxation enthalpy (Δ*H*_rel_): the aged sample showed complete relaxation (Δ*H*_rel_ = 0 kJ/mol) after 10-h annealing, representing a significant decrease from the as-cast state’s Δ*H*_rel_ of 0.442 kJ/mol. Notably, UV treatment effectively restored the relaxation enthalpy to 0.337 kJ/mol (Figure 2b). The relaxation enthalpy serves as a quantitative descriptor of atomic-scale activity within amorphous structures. The increased relaxation enthalpy is closely related to the enhanced atomic mobility and configurational excitation, providing direct thermodynamic evidence for the energy state recovery of aged MGs and the accompanying reactivation of chemical activity [38,39].

To further investigate the chemical properties of these materials, we performed comprehensive electrochemical characterization on all three sample states. The curves from corrosion potential measurements are shown in Figure 2d. After extracting the corrosion potentials of three of these samples, it can be observed that the aged samples exhibit the highest corrosion potential, followed by the as-cast samples, while the UV samples show the lowest value (Figure 2e). This inverse correlation between corrosion potential and chemical activity indicates that UV treatment significantly enhances the material’s electrochemical reactivity, with the treated samples becoming most active, followed by as-cast and then aged samples. The aged samples, characterized by lower energy states and reduced relaxation enthalpy (Δ*H*_rel_ = 0 kJ/mol), demonstrate decreased chemical activity. Conversely, UV treatment elevates the energy state and increases relaxation enthalpy (Δ*H*_rel_ = 0.337 kJ/mol), resulting in enhanced chemical reactivity. This energy-state-dependent activity is further corroborated by electrochemical impedance spectroscopy (EIS) measurements (Figure 2f), which show charge transfer resistance (Rct) values of 130 Ω, 280 Ω, and 105 Ω for as-cast, aged, and rejuvenated samples, respectively. The remarkably reduced Rct in UV samples (19% lower than as-cast and 63% lower than aged) indicates significantly improved charge transfer capability. Enhanced electron mobility at the electrode-electrolyte interface. More favorable kinetics for electrochemical reactions.

Our electrochemical characterization further demonstrates the significant enhancement in catalytic performance achieved through UV treatment. The electrochemical surface area (ECSA) measurements reveal that while the as-cast and aged samples show comparable active areas, the UV sample exhibits more than twice the ECSA of its counterparts, directly evidencing its substantially improved electrochemical reactivity (Figure S1). This remarkable increase in active surface area correlates with superior catalytic performance, as particularly demonstrated in hydrogen evolution reaction HER tests. The UV sample exhibits a significant reduction in overpotential by 229.3 mV compared to both as-cast and aged samples, indicating a notable improvement in catalytic efficiency (Figure S2). These results consistently demonstrate that the high-energy state induced by UV treatment not only enhances the material’s intrinsic activity but also creates more electrochemically active sites, resulting in simultaneous improvements in charge transfer capability and catalytic performance. The combined ECSA and HER measurements provide compelling evidence that UV treatment effectively transforms aged MGs into highly active catalytic materials, offering new possibilities for their application in energy conversion technologies.

### 3.2. Structural Differences of Different Samples After Dealloying

**Figure 3:**
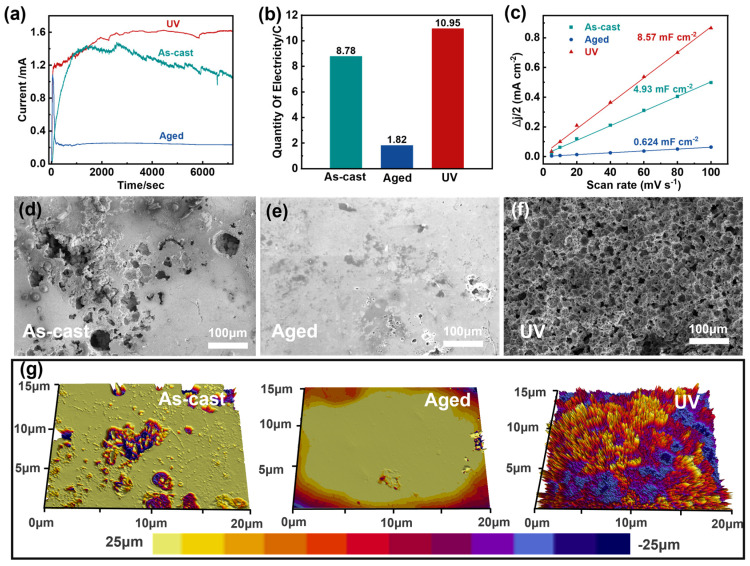
Property evolution of the samples undergoing different treatments. (**a**) For a long time dealloyed of the three samples. (**b**) Corrosive electricity of the three samples. (**c**) A linear trend of ΔJ/2 as a function of scan rate for those samples. (**d**–**f**) The SEM images of the surface of the three samples after a long-term dealloying test. (**g**) White light interference pattern of three samples.

The dealloying behavior of the three sample states was systematically investigated under controlled electrochemical conditions (2 mol/L HCl, −0.232 V vs. RHE Ag/AgCl for 2 h). The corrosion current profiles (Figure 3a) reveal distinct dissolution kinetics: while the aged sample showed rapid current decay to below 0.5 mA due to passivating oxide formation, the UV sample maintained substantially higher currents than both as-cast and aged counterparts. The corrosion charge data further corroborate these findings, providing quantitative evidence for the observed electrochemical behavior (Figure 3b).

Microstructural analysis of dealloying surfaces (Figure 3d–f) demonstrates the critical impact of pretreatment on porosity evolution: As-cast samples developed irregular, non-uniform pore structures (Figure 3d). Aged samples displayed minimal etching with random pitting (Figure 3e). UV samples achieved homogeneous nanoporous architectures (Figure 3f). Optical microscopy and higher-magnification scanning electron microscopy (SEM) images provide further corroborating evidence for these structural characteristics (Figures S3 and S4). Furthermore, as shown in Figure S4, the pores are relatively uniformly distributed within the longitudinal UV action zone.

The superior dealloying performance of rejuvenated samples originates from enhanced atomic mobility, which facilitates selective dissolution. These results demonstrate that UV treatment not only reactivates aged MGs but also enables the fabrication of high-quality porous structures that are unattainable through conventional processing, thereby opening new avenues for catalytic and functional applications.

To gain deeper insights into the dealloying mechanisms, we conducted comprehensive elemental analysis of the corroded surfaces using energy-dispersive X-ray spectroscopy (EDS). The original alloy composition (Zr_62_Cu_15.5_Ni_12.5_Al_10_) underwent significant transformation during electrochemical processing, revealing distinct elemental redistribution patterns that varied markedly with sample pretreatment. The aged samples exhibited increased zirconium content due to passivation-dominated behavior, while both as-cast and UV samples showed zirconium depletion, with the most severe reduction observed in the UV sample (from 62% to 41%). In contrast, copper displayed an opposite trend: aged samples demonstrated decreased copper content, whereas as-cast and treated samples showed copper enrichment, particularly pronounced in the UV sample where copper content surged from 15.5% to 40%. Nickel content remained relatively stable across all samples, while aluminum exhibited complete dissolution in treated regions and significant depletion in as-cast samples, consistent with its position as the most active element in the alloy system.

The EDS analysis revealed particularly striking surface composition changes in the UV samples, where distinct copper-rich zones formed (Figure S5). For the sake of comparison, we have summarized the EDS results of the samples after dealloying in a table, as shown in Table 1. This phenomenon results from the synergistic effects of preferential dissolution of more active elements (aluminum and zirconium) coupled with surface accumulation of copper atoms. The observed Zr-Cu anti-correlation demonstrates the competing dissolution-redeposition processes occurring during dealloying, while the aluminum depletion hierarchy (UV treated > as-cast > aged) clearly reflects the energy-state-dependent reactivity scale established by different pretreatments. These elemental redistribution patterns provide atomic-scale evidence that UV treatment not only lowers the activation barrier for selective dissolution but also enhances the surface diffusion of copper atoms, promoting thermodynamic equilibrium at the electrolyte interface and creating self-organized, Cu-rich surfaces with potential catalytic applications. The findings establish a quantitative framework for designing tailored surface compositions through controlled dealloying of energy-state-engineered MGs, offering new possibilities for functional material development.

To further characterize the corrosion morphology, we performed white light interferometry on the dealloying surfaces (Figure 3g). The as-cast sample exhibited irregular protrusions and depressions, confirming non-uniform corrosion. In contrast, the aged sample showed a relatively flat surface with minimal etching features. In striking contrast, the UV sample displayed a highly ordered, uniformly porous morphology with regular height variations, demonstrating superior dealloying effectiveness. This well-defined porous architecture directly results from the high-energy state achieved through UV treatment, which promotes homogeneous dissolution during the dealloying process.

The ECSA measurements (Figure 3c) revealed a remarkable enhancement in the UV samples after dealloying, showing an over 200-fold increase compared to their pre-dealloying state (Figure S6). This dramatic improvement in ECSA provides quantitative evidence of successful porous structure formation and confirms the critical advantage of UV pretreatment in creating catalytically active surfaces. The combination of interferometry and ECSA results establishes a clear correlation between the uniform porous morphology and exceptional electrochemical performance, highlighting the importance of energy-state engineering through UV treatment for developing high-performance catalytic materials [40]. These findings demonstrate that the synergistic effect of UV pretreatment and controlled dealloying can effectively transform monolithic MGs into functional porous materials with outstanding catalytic potential.

### 3.3. Catalytic Performances of Different Samples After Dealloying

**Figure 4:**
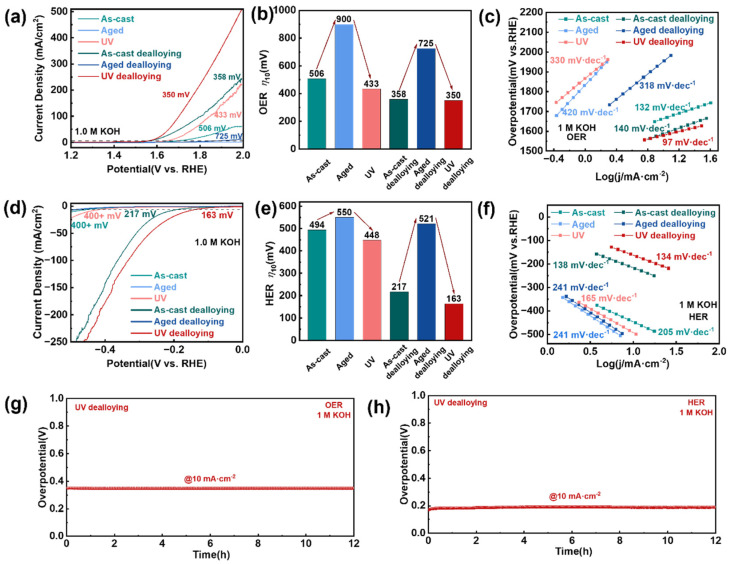
HER and OER electrocatalytic property characterization. (**a**) The HER polarization curves for the three samples, before and after dealloying, were acquired using linear sweep voltammetry (LSV) with a scan rate of 0.5 mV s^−1^ in 1 M KOH at room temperature. (**b**) 10 mV·cm^−2^ overpotential bar chart form (**a**). (**c**) Corresponding Tafel slope derived from (**a**) where the Tafel slope is identified. (**d**) The OER polarization curves for the three samples, before and after dealloying, were acquired using linear sweep voltammetry with a scan rate of 0.5 mV s^−1^ in 1 M KOH at room temperature. (**e**) 10 mV·cm^−2^ overpotential bar chart form (**d**). (**f**) Corresponding Tafel slope derived from (**d**) where the Tafel slope is identified. (**g**,**h**) Stability tests: chronopotentiometry curves at constant current density of 10 mA/cm^2^ for OER and HER.

The catalytic performance evaluation in alkaline media (1 M KOH) revealed that the UV treatment followed by the dealloyed sample exhibited exceptional electrocatalytic activity for both OER and HER. This optimized sample achieved remarkably low overpotentials of 350 mV for OER (Figure 4a) and 163 mV for HER (Figure 4d), representing the best performance among all tested samples, including as-cast, aged, and their dealloyed counterparts. While the dealloyed as-cast sample showed a comparable OER overpotential of 358 mV, the UV sample demonstrated superior catalytic efficiency at higher current densities, highlighting the advantage of its unique microstructure. The catalyst also demonstrates commendable performance in the HER under acidic conditions, exhibiting competitive activity and stability compared to established benchmark materials (Figure S7). The consistent trend observed in overpotential measurements, where UV treatment lowered the overpotential while aging increased it, was evident for both non-dealloyed and dealloyed samples in both OER and HER tests (Figure 4b,e), confirming the crucial role of energy state modulation in enhancing catalytic performance.

Kinetic analysis through Tafel slopes further validated the improved catalytic behavior, with the UV treatment and dealloyed sample showing the most favorable reaction kinetics (97 mV·dec^−1^ for OER and 134 mV·dec^−1^ for HER) in 1 M KOH (Figure 4c,f). The sample’s excellent durability was confirmed through a 12-h stability test at 10 mA·cm^−2^, which revealed only minimal potential variation, demonstrating robust structural integrity under prolonged operation (Figure 4g,h). Comparative analysis of the hydrogen and oxygen evolution polarization curves before and after stability testing reveals minimal performance degradation, with only a marginal increase in overpotential of 1.2 mV for the OER and 10.4 mV for the HER at a current density of 10 mA cm^−2^ (Figure S8). Furthermore, the ECSA experienced merely a slight reduction after the durability test, further attesting to the exceptional structural and catalytic stability of the material. These results collectively demonstrate the outstanding durability of this catalyst under operational conditions (Figure S9). This outstanding catalytic performance stems from the synergistic combination of several factors: the high-energy state induced by UV treatment, which enhances charge transfer efficiency; the uniform porous structure, providing abundant active sites; and the copper-enriched surface, which optimizes the adsorption of reaction intermediates [41,42,43]. The results establish that energy-state engineering through UV treatment, combined with controlled dealloying, can effectively transform MGs into high-performance, durable electrocatalysts for water splitting applications, offering a promising alternative to conventional precious metal-based catalysts.

### 3.4. Comparison of Catalytic Performances

**Figure 5:**
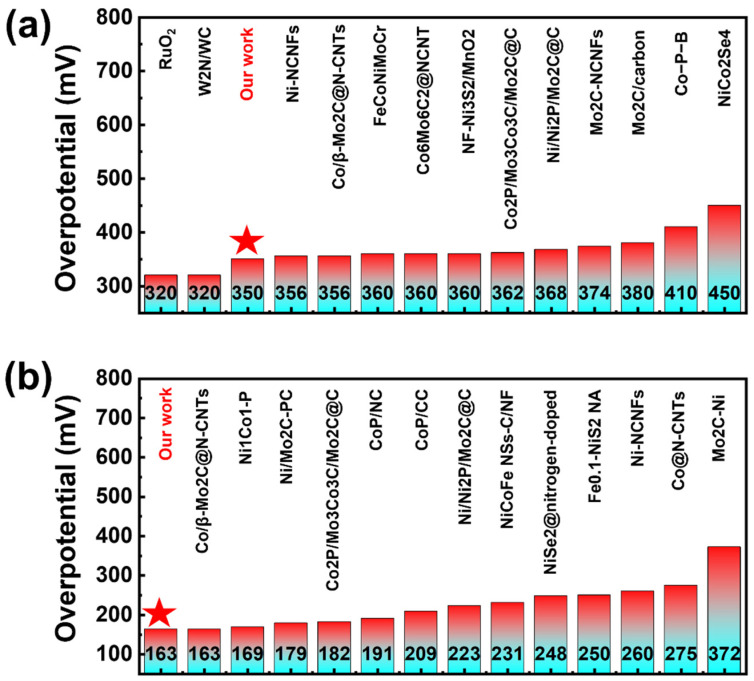
The overpotential *η*_10_, water splitting of UV dealloying in OER and HER reactions were compared with those reported in the literature for electrocatalysts. The stars represent our materials. (**a**) Overpotential of oxygen evolution reaction (OER) for different non-metallic catalysts in the literature and RuO_2_ under a current density of 10 mA/cm^2^. (**b**) Overpotential of hydrogen evolution reaction (HER) for different non-metallic catalysts in the literature under a current density of 10 mA/cm^2^.

As a type of non-noble metal catalyst, the porous catalyst prepared by this method exhibits significantly superior OER and HER performances compared to other non-noble metal catalysts reported in the literature. Notably, our material exhibits OER performance in alkaline conditions that is nearly comparable to commercial RuO_2_ benchmarks (Figure 5a) [44,45,46,47]. In terms of HER, our materials have performed quite well compared to the non-metal catalysts reported in other literature (Figure 5b).This exceptional performance positions our UV engineered porous MG as a highly competitive alternative to precious metal catalysts in alkaline electrolysis systems. The combination of non-noble metal-like activity with the inherent advantages of MGs—including tunable composition, scalable fabrication, and structural homogeneity—suggests great potential for practical applications. The achieved performance metrics, especially when considering the material’s non-precious nature and excellent durability demonstrated in long-term testing, represent a significant advancement in the development of cost-effective, high-performance electrocatalysts for water splitting technologies. These results highlight how energy-state engineering through UV treatment, combined with optimized dealloying protocols, can bridge the performance gap between conventional non-noble catalysts and precious metal benchmarks, opening new possibilities for sustainable hydrogen production.

## 4. Conclusions

In this study, we report that the AAUV treatment promotes the formation of a uniform porous structure during the dealloying process of MGs. This energy-state modulation engineering enhances the atomic mobility and chemical activity of samples subjected to UV treatment, thereby effectively reducing the kinetic barriers associated with selective dissolution during dealloying. The dealloyed porous samples prepared via this method exhibit excellent electrochemical performance. Under alkaline conditions, these materials achieve outstanding catalytic performance: at a current density of 10 mA/cm^2^, the overpotentials for the OER and HER are only 350 mV and 163 mV, respectively. This performance places the materials among the top-performing non-precious metal catalysts for these reactions. These findings demonstrate that UV treatment is a promising technique for the fabrication and modification of porous MGs, holding significant potential for broad practical applications.

## Figures and Tables

**Table 1:** Elemental compositions of the samples after dealloying.

Sample	Zr (%)	Cu (%)	Ni (%)	Al (%)
As-cast	70	12	8	10
Aged	74	8	8	10
UV	40	41	13	6

## Data Availability

The original contributions presented in this study are included in the article/Supplementary Materials. Further inquiries can be directed to the corresponding authors.

## Supplementary Materials

The following supporting information can be downloaded at: https://www.mdpi.com/article/10.3390/ma18245484/s1. Figure S1: The CV curve and ECSA in 0.5 M H_2_SO_4_ solution; Figure S2: The HER tests and overpotential of the three samples; Figure S3: Optical photograph of the three samples after dealloying; Figure S4: The SEM images of the samples after dealloying; Figure S5: SEM and elemental content characterization; Figure S6: CV curves in 0.5 M H_2_SO_4_ solution; Figure S7: Performance test of HER in 0.5 M H_2_SO_4_; Figure S8: LSV curves before and after the Stability tests; Figure S9: The CV curve and ECSA in 0.5 M H_2_SO_4_ solution.
