# Supplementary material for: Preparation and Application of Porous Metallic Glasses via Aging-Assisted Ultrasonic Vibration and Compression
Source: Materials (Basel). 2025 Dec 5;18(24):5484. doi: 10.3390/ma18245484 (PMC12734923; doi:10.3390/ma18245484)
Supplement: Supplementary file 1 [file materials-18-05484-s001.zip › materials-4005033-supplementary.pdf]

Supplementary Materials for

Preparation and Application of Porous Metallic Glasses via Aging-Assisted Ultrasonic Vibration and Compression

Jiaqing Lin [1,†], Heting Zhang [1,†], Zhe Chen [1,2], Jihan Jiang [1], Xingran Zhao [1], Xiaodi Liu [1,*], Wenqing Ruan [1,*] and Jiang Ma [1,*]

[1] *Shenzhen Key Laboratory of High Performance Nontraditional Manufacturing, College of Mechatronics and Control Engineering, Shenzhen University, Shenzhen 518060, China.*

[2] *School of Materials Science and Engineering, Central South University, 932 South Lushan Road, Changsha 410083, China*

[†] *These authors contributed equally to this work.*

* *Corresponding authors.*

E-mail addresses: xdliu2018@szu.edu.cn (X.L.); ruanwq@szu.edu.cn (W.R.); majiang@szu.edu.cn (J.M.)

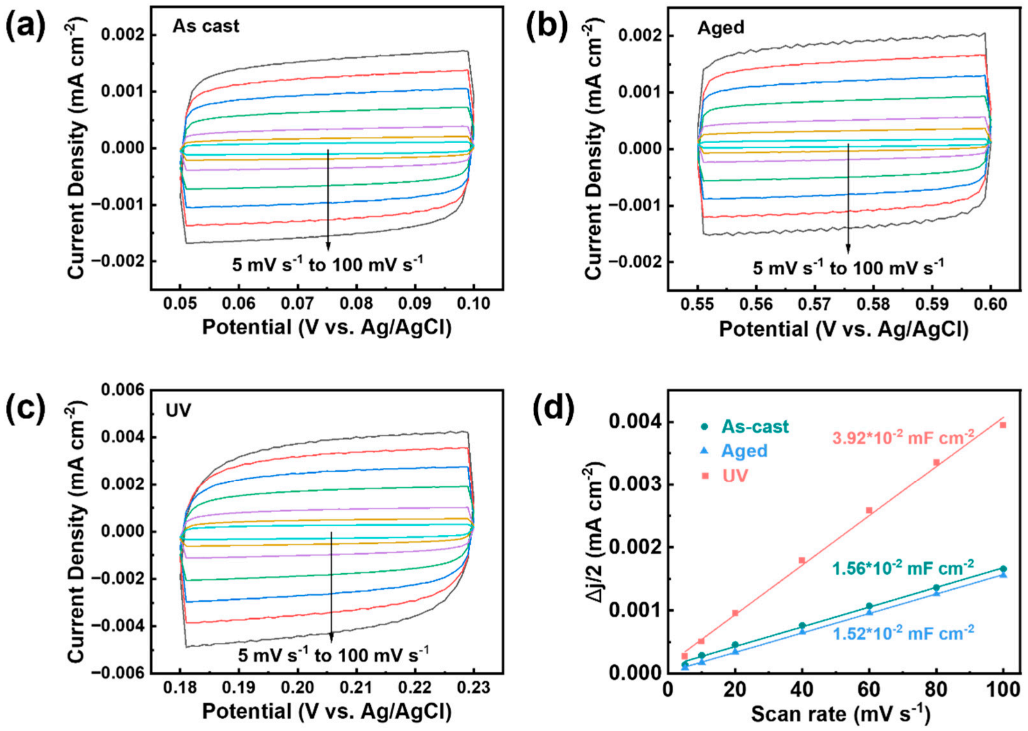

Figure. S1. The CV curve and ECSA in 0.5 M H_2SO_4 solution. (a) As-cast, (b) Aged, (c) UV show the double layer capacitance without electrochemical reactions. (d) A linear trend of $\Delta J/2$ as a function of scan rate for the three samples.

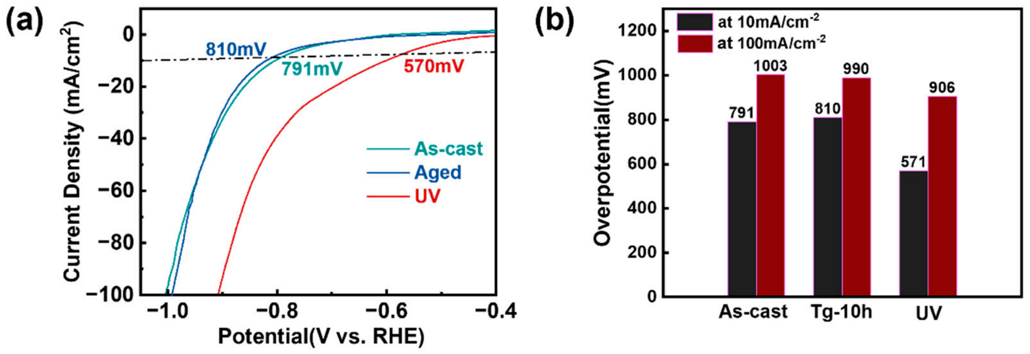

Figure. S2. The HER tests and overpotential of the three samples. (a) Three samples were subjected to HER in 0.5M H_2SO_4. (b) 10 mA·cm^{-2}Current density and 100 mA·cm^{-2} current density overpotential bar charts.

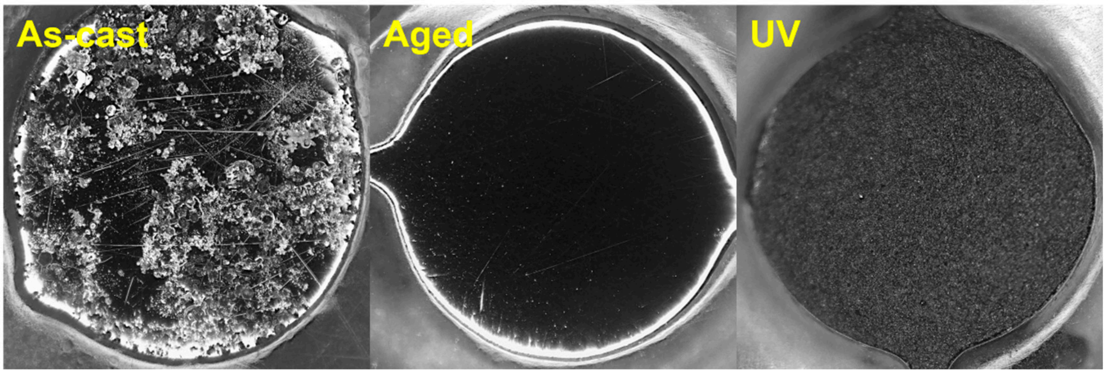

Figure. S3. Optical photograph of the three samples after dealloying

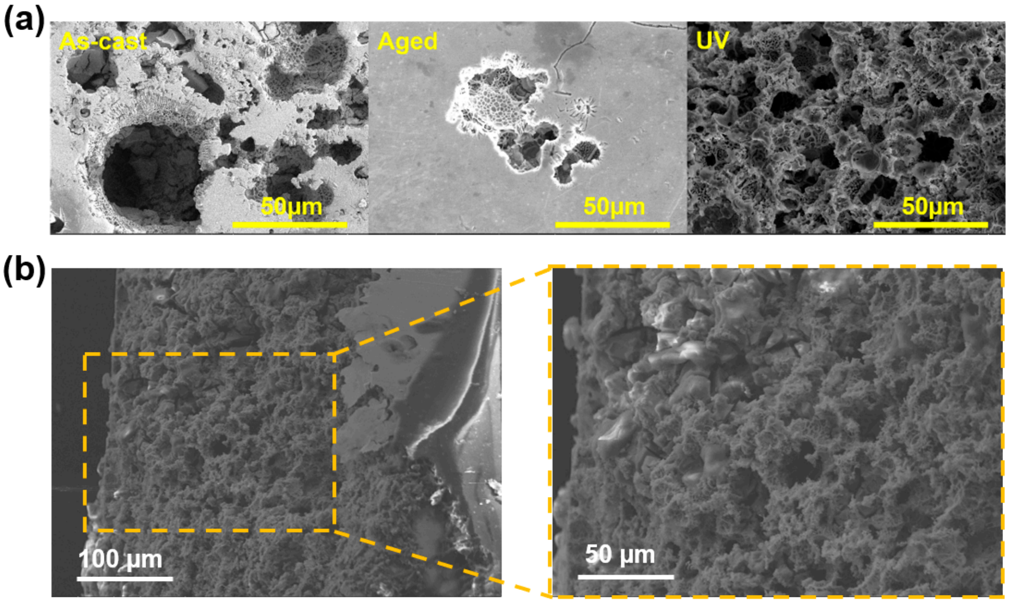

Figure. S4. The SEM images of the samples after dealloying. (a) Surface Morphologies of Different Samples (b) SEM Images of the Cross-Sections of UV treated Samples

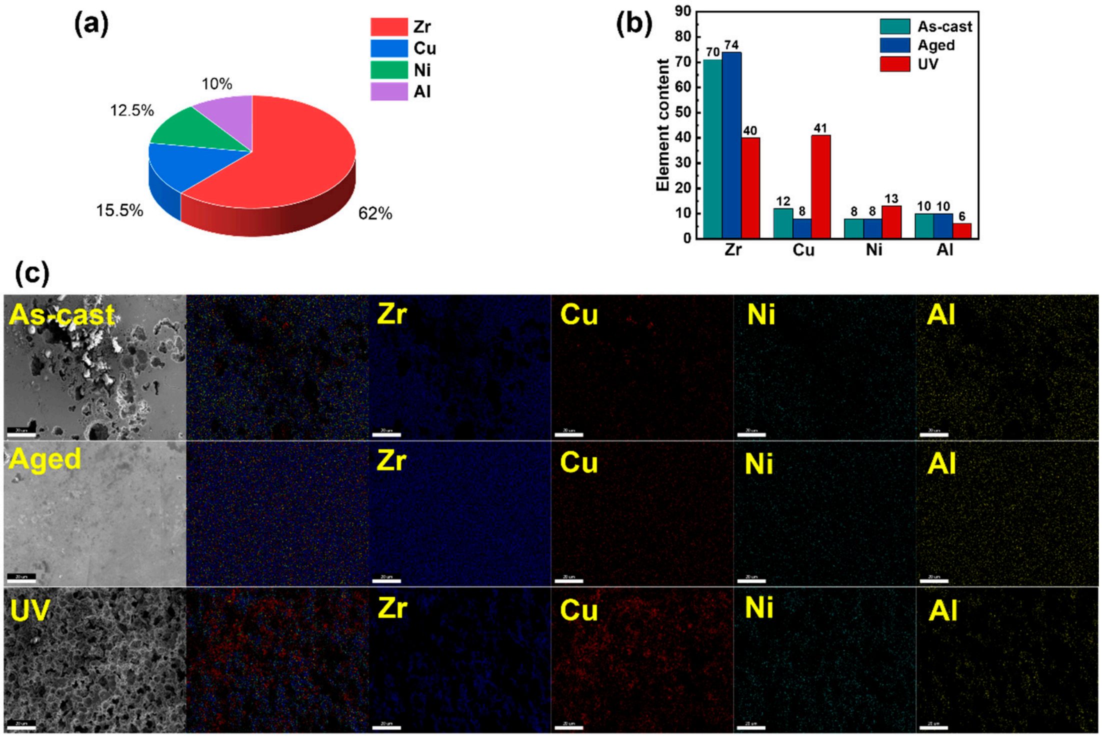

Figure. S5. SEM and elemental content characterization. (a) The initial elemental content in the as-cast state. (b) The content of elements for the three samples after dealloying (The data is sourced from c). (c) SEM and EDS of the three samples after dealloying.

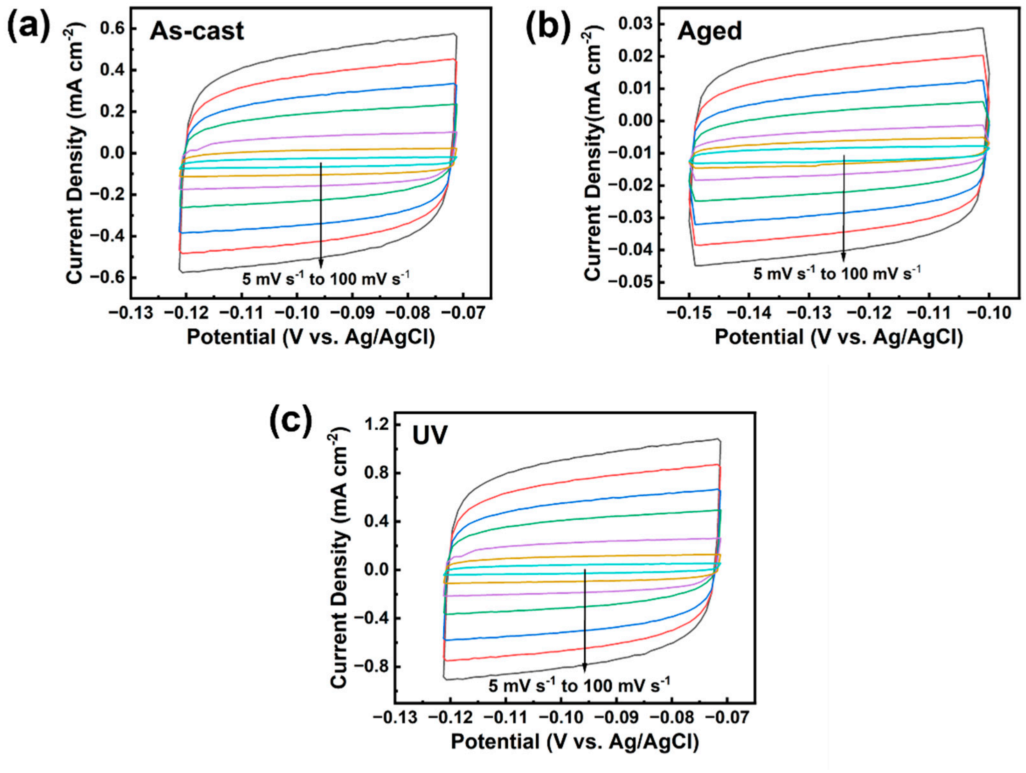

Figure. S6. CV curves in 0.5 M H_2SO_4 solution. (a) As-cast dealloying, (b) Aged dealloying, (c) UV dealloying show the double-layer capacitance without electrochemical reactions. Show the double-layer capacitance without electrochemical reactions.

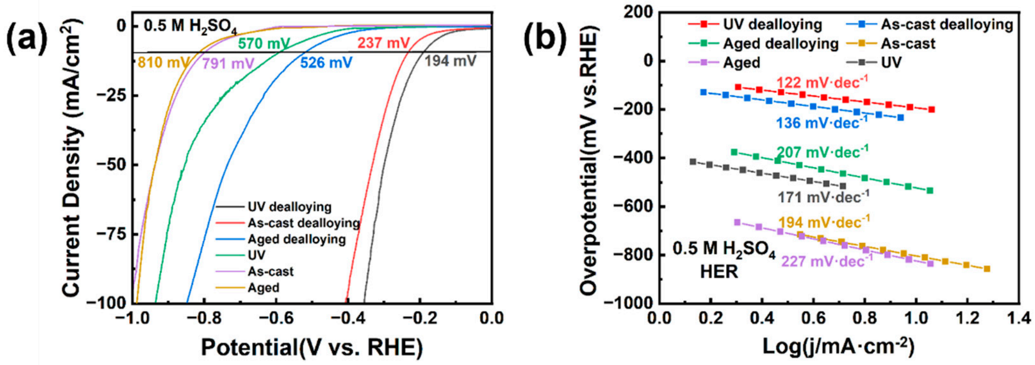

Figure. S7 Performance test of HER in 0.5 M H_2SO_4. (a) The HER polarization curves for the three samples before and after dealloying acquired by linear sweep voltammetry (LSV) with a scan rate of 0.5 mV s^{-1} in 0.5 M H_2SO_4 at room temperature. (b) Corresponding Tafel slope derived from a) where the Tafel slope is identified.

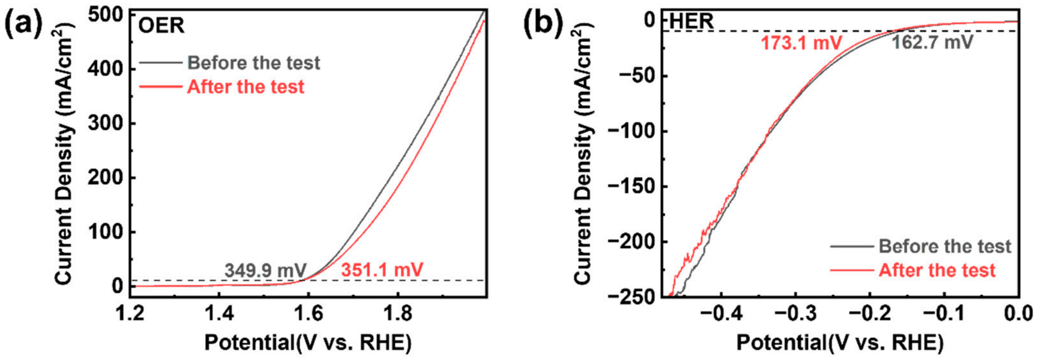

Figure. S8 LSV curves before and after the Stability tests. (a) OER curves. (b) HER curves.

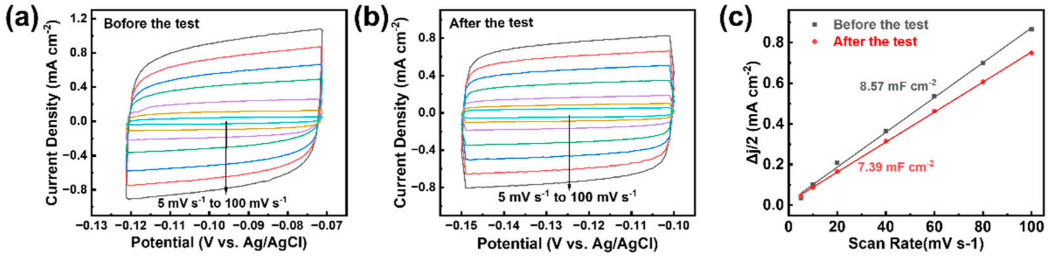

Figure. S9. The CV curve and ECSA in 0.5 M H_2SO_4 solution. (a) UV dealloying before the Stability tests, (b) UV dealloying after the Stability tests, (c) A linear trend of $\Delta J/2$ as a function of scan rate for the two samples.
